# Astrocytes and radial glia‐like cells, but not neurons, display a nonapoptotic increase in caspase‐3 expression following exercise

**DOI:** 10.1002/brb3.1110

**Published:** 2018-09-21

**Authors:** Morgan E. Stevenson, Nicole A. Lensmire, Rodney A. Swain

**Affiliations:** ^1^ Department of Psychology University of Wisconsin‐Milwaukee Milwaukee Wisconsin

**Keywords:** Cleaved caspase‐3, Neurogenesis, Nonapoptotic, Plasticity, Radial glia

## Abstract

**Background:**

Exercise induces plasticity in the hippocampus, which includes increases in neurogenesis, the proliferation of new neurons, and angiogenesis, the sprouting of new capillaries from preexisting blood vessels. Following exercise, astrocytes also undergo morphological changes that parallel the events occurring in the neurovascular system. Interestingly, there have also been reports of apoptosis in the hippocampus following aerobic exercise. This experiment aimed to identify which population of hippocampal cells undergoes apoptosis after an acute bout of exercise.

**Methods:**

Cleaved caspase‐3, a terminal protein in the apoptotic cascade, was initially used to identify apoptotic cells in the hippocampus after rats completed an acute bout of exercise. Next, the proportion of immature neurons, adult neurons, astrocytes, or radial glia‐like cells expressing cleaved caspase‐3 was quantified. TUNEL staining was completed as a second measure of apoptosis.

**Results:**

Following exercise, cleaved caspase‐3 expression was increased in the CA1 and DG regions of the hippocampus. Cleaved caspase‐3 was not highly expressed in neuronal populations, and expression was not increased in these cells postexercise. Instead, cleaved caspase‐3 was predominantly expressed in astrocytes. Following exercise, there was an increased number of cleaved caspase‐3 positive astrocytes in DG and CA1, and cleaved caspase‐3 positive radial glia‐like cells located in the subgranular zone. To determine whether cleaved caspase‐3 expression in these glial cells was associated with apoptosis, a TUNEL assay was completed. TUNEL staining was negligible in all groups and did not mirror the pattern of caspase‐3 labeling.

**Conclusions:**

Cleaved caspase‐3 expression was detected largely in non‐neuronal cell populations, and the pattern of cleaved caspase‐3 expression did not match that of TUNEL. This suggests that after exercise, cleaved caspase‐3 expression may serve a nonapoptotic role in these hippocampal astrocytes and radial glia‐like cells. It will be important to identify the function of exercise‐induced cleaved caspase‐3 expression in the future experiments.

## 1 INTRODUCTION

Aerobic exercise produces plasticity in the brain. In the hippocampus, exercise accelerates neurogenesis, the proliferation of new neurons in the subgranular zone (SGZ) of the dentate gyrus (DG; van Praag, Kempermann, & Gage, 1999; van Praag, Shubert, Zhao, & Gage, 2005). Exercise also increases the density of dendritic spines (Dietrich, Andrews, & Horvath, 2008; Stranahan, Khalil, & Gould, 2007), and elevates angiogenesis, the birth of new vessels from preexisting capillaries, in the hippocampus (Kerr & Swain, 2011; Kerr, Steuer, Pochtarev, & Swain, 2010; Van der Borght et al., 2009). In addition to plasticity in neurons and vessels, astrocytes are also modified by exercise (Brockett, LaMarca, & Gould, 2015; Ferreira, Real, Rodrigues, Alves, & Britto, 2011; Komitova, Zhao, Gidö, Johansson, & Eriksson, 2005; Li et al., 2005; Saur, et al., 2014; Uda, Ishido, Kami, & Masuhara, 2006). In DG, seven days of exercise increases the proliferation of glial fibrillary acidic protein (GFAP) positive cells (Komitova et al., 2005; Uda et al., 2006), and just three days of exercise increases astrocytic GFAP expression in the hilar region (Ferreira et al., 2011). The precursor cells that give rise to new neurons in the hippocampus are GFAP and vimentin (an intermediate filament protein expressed in immature astrocytes and radial glia) positive and share characteristics with astrocytes and radial glia (Filippov et al., 2003; Garcia, Doan, Imura, Bush, & Sofroniew, 2004; Gubert, Zaverucha‐do‐Valle, Pimentel‐Coelho, Mendez‐Otero, & Santiago, 2009; Kempermann, 2015; Kriegstein & Alvarez‐Buylla, 2011; Seri, Garcia‐Verdugo, McEwen, & Alvarez‐Buylla, 2001; Song, Stevens, & Gage, 2002). Based on their shared characteristics with glia, these neuronal precursor cells are often termed radial astrocytes or radial glia‐like (RGL) cells (Filippov et al., 2003; Gebara et al., 2016; Kempermann, 2015; Kriegstein & Alvarez‐Buylla, 2011). Approximately one month of treadmill exercise increases the density of GFAP positive astrocytes in CA1 (Rodrigues et al., 2010; Saur et al., 2014). Exercise does not alter astrocyte number in CA1, but astrocytic processes tend to be longer in length in exercising animals (Saur et al., 2014). This elongation of astrocytic processes may be linked to increases in dendritic spine density also associated with exercise (Stranahan et al., 2007; Saur et al., 2014). Conversely, others have found no change or decreases in GFAP expression between exercise and sedentary animals in CA1 (Bernardi et al., 2013; de Senna et al., 2011). Despite some variability in results, evidence abundantly indicates aerobic exercise stimulates the growth of vessels, neurons, and astrocytes in the hippocampus (Saur et al., 2014; Uda et al., 2006; Van der Borght et al., 2009; van Praag et al., 1999; van Praag et al., 2005). These events increase hippocampal volume and, in turn, support exercise‐induced neuroprotection, recovery from insult, and learning and memory improvements (Austin, Ploughman, Glynn, & Corbett, 2014; Colcombe et al., 2006; Erickson et al., 2009, 2011; Pohlack et al., 2014).

Mixed findings regarding hippocampal apoptotic cell death after exercise have also been reported (Kerr & Swain, 2011; Kim et al., 2002; Kitamura & Sugiyama, 2006; Li, Liu, & Yan, 2013). Seven days of treadmill running increases cell proliferation, but not apoptosis, in the rat DG (Kim et al., 2002); however, 7–21 days of wheel running increases both cell proliferation and apoptosis in the mouse DG (Kitamura & Sugiyama, 2006). In the later experiment, the death of adult, granule cell layer neurons was enhanced, and Kitamura and Sugiyama (2006) propose that exercise may accelerate the rate at which adult neurons are replaced by newly generated neurons. In other paradigms, elevated apoptosis in the hippocampus has also been documented after just a single day of wheel or treadmill running (Kerr & Swain, 2011; Li et al., 2013). Inconsistencies in results may relate to variations in the exercise parameters and apoptosis quantification methods used across experiments. Markers of apoptotic cell death include cleaved caspase‐3, a terminal protein in the apoptotic cascade inducing irreversible fragmentation of DNA (Nakajima & Kuranaga, 2017; Wolf, Schuler, Echeverri, & Green, 1999), staining DNA breakages using a terminal deoxynucleotidyl transferase (TdT) dUTP Nick‐End Labeling (TUNEL) assay, and analyzing the ratio of pro‐apoptotic molecules such as Bax to anti‐apoptotic molecules like Bcl‐2 (Li et al., 2013).

Based on findings that suggest an acute, 24 hr, bout of exercise increases apoptosis in the hippocampus, our hypothesis initially was that some cells are unable to adapt to the increased metabolic demands of aerobic exercise and thus fail to survive. However, beyond a few reports of cell death, no studies were found that identified which cells express these apoptotic markers after a single bout of exercise. Because this effect has been found in the hippocampus, our first aim was to use cleaved caspase‐3 to identify what regions of the hippocampus displayed elevated apoptotic cell death following exercise: CA1, CA2/3, or DG. After showing caspase‐3 was elevated in CA1 and DG regions of exercising animals, our second aim was to identify whether newly birthed neurons in DG, or adult neuronal populations present in both CA1 and DG were disproportionately affected by apoptotic cell death. Interestingly, we found cleaved caspase‐3 was not highly expressed in either neuronal population, but instead in nonapoptotic astrocytes and radial glia‐like cells. This effect was observed in CA1 and DG, regions of the hippocampus where aerobic exercise produces structural plasticity in astrocytes (Ferreira et al., 2011; Komitova et al., 2005; Rodrigues et al., 2010; Saur et al., 2014; Uda et al., 2006).

## 2 METHODS

### 2.1 Animals

Twenty, male Long Evans hooded rats (*Rattus norvegicus*; 175–200 g,) were purchased from Envigo (Madison, WI) and randomly divided into two equal groups: Inactive Control (IC; *n = *10) and Voluntary Exercise (VX; *n = *10). Upon arrival, all animals were housed individually and allowed 1 week to acclimate prior to beginning experimental procedures. Animals were housed on a 12‐hr light/dark cycle and food and water were available ad libitum throughout the duration of the experiment. All procedures were approved by the Institutional Animal Care and Use Committee (IACUC) at the University of Wisconsin‐Milwaukee.

### 2.2 Exercise procedure

After the one‐week acclimation, each VX animal was moved to a cage that contained an in‐cage running wheel. For all running wheels, one revolution equated to ~1 m. The VX animals were housed individually in these cages for 24 hr and had unrestricted access to the running wheels. During this 24‐hr exercise bout, IC animals remained sedentary in their home cages. Upon completion of this acute bout of exercise, the distance run by each VX animal was recorded, and all animals were euthanized.

### 2.3 Tissue preparation

Animals were sacrificed by immersion in a carbon dioxide tank. Brains were then removed, rapidly frozen in chilled isopentane and stored at −80°C until sectioning. Prior to sectioning, all brains were hemisected. Each hemisphere was sectioned coronally at 12 μm using a Leica CM 3050 S cryostat (Wetzlar, Germany), and sections were collected through the entire dorsal hippocampus beginning –1.90 mm from Bregma and ending –3.90 mm (Praxinos & Watson, 1998). For each animal, twenty slides were collected, and, on each slide, a total of eight tissue sections were mounted. These eight sections were spaced 240 μm apart and were representative of the entire dorsal hippocampus. One slide per animal was randomly selected for each later analysis.

### 2.4 Caspase‐3 expression in hippocampal regions

This step aimed to identify which regions of the hippocampus (CA1, CA2/3, DG) displayed differences in cleaved caspase‐3 expression based on exercise condition. For a subset of animals (*n = *5 per group), one slide per animal was prepared for an immunohistochemistry (IHC) procedure labeling cleaved caspase‐3. See Table 1 for information regarding the purchase and dilution of all antibodies. Tissue was first fixed by immersion in chilled acetone (–20°C) for 10 min. Slides dried for 20 min before beginning IHC. Once the tissue was dry, all slides went through a series of phosphate‐buffered saline (PBS) washes. Peroxidase activity was blocked with a 10 min wash in 0.3% hydrogen peroxide in PBS, and tissue was again washed in PBS. Slides were incubated for 1 hr in a blocking solution containing 10% goat serum, 0.5% Triton X (10% concentration) and PBS at room temperature. After draining off the blocking solution, the cleaved caspase‐3 antibody solution was applied to each slide and tissue was incubated overnight at 4°C. The next day, the primary antibody solution was drained off the slides, and slides were washed in PBS. Tissue was then incubated in the biotinylated anti‐rabbit secondary antibody solution for 1 hr and 30 min in a humid chamber at room temperature. Following this incubation, the secondary solution was drained off of slides, and tissue was again washed in PBS. Next, the avidin biotin complex (Vector Laboratories, Burlingame, CA) was pipetted onto all slides and tissue incubated in a humid chamber for 1 hr at room temperature. Finally, tissue was washed with PBS, reacted in 3–3′ diaminobenzidine (DAB; Millipore Sigma), mounted using Permount (Thermo Fisher Scientific) medium, and coverslipped. One section per animal went through the IHC procedure but was not treated with the primary antibody, serving as a negative control.

**Table 1:** Antibodies

Antibody	Source	Dilution	Company Name/Catalog Number
Anti‐Cleaved Caspase‐3	Rabbit	1:400	Cell Signaling Technology/9661
Anti‐NeuN	Mouse	1:500	Millipore Sigma/MAB377
Anti‐Doublecortin	Mouse	1:100	Santa Cruz Biotechnology, Inc/sc−271390
Anti‐GFAP	Mouse	1:400	Santa Cruz Biotechnology, Inc/sc−33673
Anti‐Vimentin	Mouse	1:100	Santa Cruz Biotechnology, Inc/sc−6260
Biotinylated Goat Anti‐Rabbit IgG Antibody	Goat	1:250	Vector Laboratories/BA−1000
Goat Anti‐Mouse IgG1 Secondary Antibody, Alexa Fluor 594	Goat	1:250	Thermo Fisher Scientific/A−21125
Goat Anti‐Rabbit IgG (H + L) Highly Cross‐Adsorbed Secondary Antibody, Alexa Fluor 488	Goat	1:250	Thermo Fisher Scientific/A−11034
Goat Anti‐Mouse IgG (H + L) Highly Cross‐Adsorbed Secondary Antibody, Alexa Fluor 350	Goat	1:250	Thermo Fisher Scientific/A−21049

Tissue was imaged using a light microscope (Olympus, America, Inc., Center Valley, PA) with an attached SPOT digital camera (Diagnostic Instruments, Inc., Sterling Heights, MI). Five sections per animal were randomly selected for imaging at 400× magnification. Nonoverlapping images were taken so the entire dorsal hippocampus was captured on each section. After tissue sections were imaged, unbiased stereology was used to quantify the total area fraction of caspase‐3 labeling in each region of the hippocampus. Experimenters were blind to the animal's exercise condition during quantification. For quantification, the hippocampus was divided into three regions: CA1, CA2/3, and DG. Analysis of the DG region always included the hilus, SGZ, granule cell layer, and molecular layer. Images from each animal were randomly selected for unbiased stereology using the point counting technique described by Gunderson, Jensen, Kiêu, and Nielsen (1998). Once an image was selected, it was imported into ImageJ software (US National Institutes of Health, Bethesda, MD, https://imagej.nih.gov/ij/) and converted into an 8‐bit image. Pixel intensity was set automatically by selecting the maximum entropy threshold method. A point grid was then superimposed, and if the point fell on caspase‐3 labeling, it was counted, and area fractions were calculated for each region of the hippocampus (CA1, CA2/3, DG) by dividing the total number of points that fell on labeling by the total number of points on the grid. This analysis provided the relative area of cleaved caspase‐3 expression for each animal, in each region of the hippocampus.

### 2.5 Immunofluorescence labeling by cell type

Once the regions of the hippocampus with an elevated number of cleaved caspase‐3 positive cells following exercise were identified, we next determined which cell types were expressing caspase‐3 in these regions. Immunofluorescence (IF) procedures were completed to double‐label cleaved caspase‐3 and antigens present in the differing cell types. Mature neurons were labeled using neuronal nuclear protein (NeuN), immature neurons were identified using doublecortin (DCX), RGL cells were labeled using vimentin, and astrocytes were identified using GFAP. Vimentin is also expressed in large blood vessels; however, these large vessels were not included in our analyses. For each double‐labeling procedure, an additional slide was randomly selected for each animal. The IF procedure was similar to the method described previously; however, all sections were fixed in 10% neutral buffered formalin (Millipore Sigma) for 15 min and moved directly into PBS wash buffer to begin IF. All PBS washes contained 0.05% Tween‐20 (Millipore Sigma) and prior to incubation in the blocking solution, tissue was permeabilized for 15 min in 0.3% Triton X (10% concentration). Primary antibody solutions targeting either: cleaved caspase‐3 and NeuN, cleaved caspase‐3 and DCX, cleaved caspase‐3 and vimentin, or cleaved caspase‐3 and GFAP were prepared (see Table 1), and each solution was applied to one slide per animal. Tissue again incubated overnight at 4°C. Day two was identical to the procedure previously described, except the appropriate Alexa Fluor secondary antibodies were applied for 2 hr (see Table 1). Finally, slides were washed with PBS, mounted using Prolong Gold (Thermo Fisher Scientific) medium, and immediately coverslipped. For each set of IF double‐labels, one tissue section per animal was not treated with the primary antibodies and served a negative control.

Imaging and quantification were also similar to the previous description, but this time required a Nikon Eclipse (Nikon, Melville, NY) fluorescence microscope for imaging. Exposure parameters were identical across the collection of all images to allow for comparison between sections. For all caspase‐3 images, a 9‐pixel‐radius rolling‐ball subtraction algorithm was used to remove background in ImageJ software prior to quantification (Sternberg, 1983), and thresholds were set automatically using the maximum entropy algorithm. Unbiased stereology was also similar; however, this time ImageJ software was used to count only cleaved caspase‐3 labeling that colocalized, or overlapped, with NeuN, DCX, vimentin, or GFAP. This provided area fractions for caspase‐3 co‐expression with mature neurons, immature neurons, astrocytes, or RGL cells for each animal, in each region of the hippocampus.

### 2.6 TUNEL assay for apoptotic cell death

Because cleaved caspase‐3 expression was more robust than expected, a second method was used to identify apoptotic cell death and determine whether caspase‐3 expression was associated with apoptosis. An ApopTag® In Situ Apoptosis Detection Kit (Millipore Sigma) was used to label DNA fragmentation, present in apoptotic cells. The kit stained both single and double stranded breaks that are associated with apoptotic cell death using the TUNEL assay technique. The assay was completed on one slide per animal per the directions provided in the kit. Sections were reacted in a DAB horseradish peroxidase (HRP) substrate, counterstained using Safranin‐O (Millipore Sigma), mounted with Permount (Thermo Fisher Scientific) medium, and coverslipped. Imaging and unbiased stereology was identical to methods described previously for caspase‐3, and area fractions for TUNEL positive cells were calculated for each animal. One section per animal was treated with DNase1, an enzyme that nonspecifically cleaves DNA, for 10 min at room temperature and served as a positive control. One section per animal was not treated with the terminal deoxynucleotidyl transferase (TdT) enzyme and served as a negative control.

### 2.7 Statistics

For all labels of interest, an average area fraction was calculated for each rat, in each hippocampal region (CA1, CA2/3, DG). Next, area fractions for rats in the VX and IC groups were averaged across groups by hippocampal region. No outliers were identified. Two‐tailed, unpaired Student's *t*‐tests were completed for each hippocampal region (CA1, CA2/3, DG) comparing the two exercise conditions (VX or IC) on the area fractions of cleaved caspase‐3 expression, cleaved caspase‐3 expression in the various cell types (mature neurons, immature neurons, astrocytes, and RGL cells), and TUNEL labeling of apoptotic cells. All area fraction data are presented as the mean (*M*) ± Standard Error of the Mean (*SEM*). For all analyses, a *p* value of <0.05 was considered statically significant.

## 3 RESULTS

### 3.1 Exercise behavior

All voluntary exercise animals engaged with wheel running during the 24‐hr exercise period. The mean distance run was 1,200 m ± 86 m.

### 3.2 Exercise increased caspase‐3 expression in CA1 and DG

**Figure 1:**
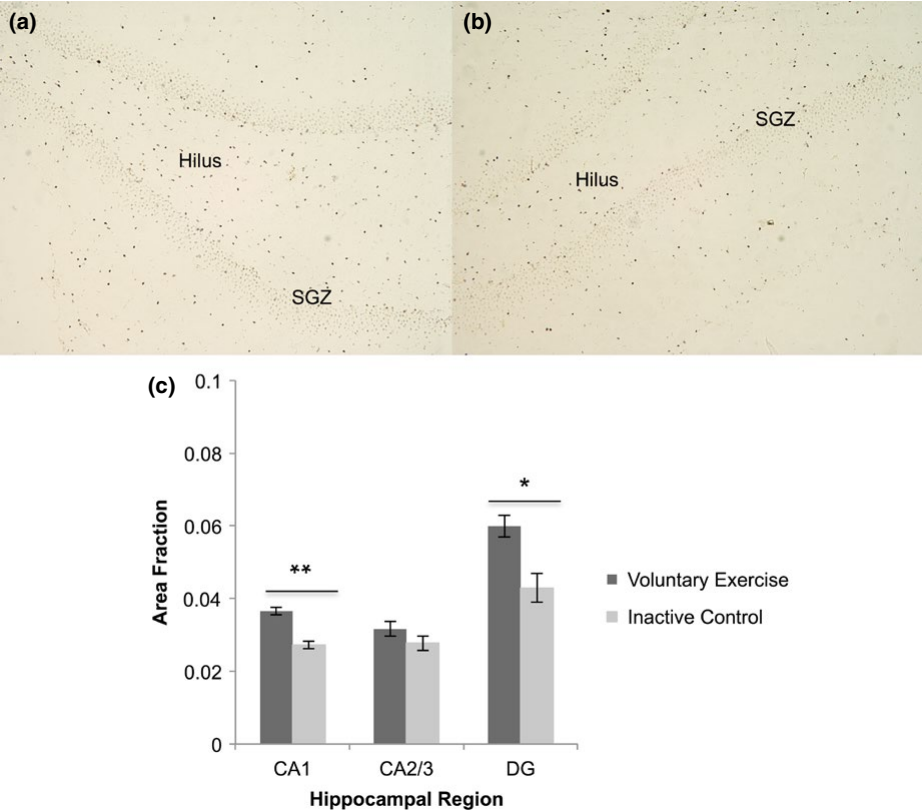
Caspase‐3 expression by hippocampal region. (a) Caspase‐3 labeling (dark brown) in the dentate gyrus (DG) of a voluntary exercise animal. (b) Caspase‐3 labeling in DG of an inactive control animal. Differences between voluntary exercise and control were most apparent in the hilus and subgranular zone (SGZ) of DG. (c) The voluntary exercise group displayed a significant elevation in caspase‐3 expression in CA1 (*t*_(8)_ = 4.98, **p < 0.01) and DG (*t*_(8)_ = 3.81, *p < 0.05) compared to inactive controls. There was no difference between groups in CA2/3 (*t*_(8)_ = 1.63, *p* > 0.05). Values are mean ± *SEM*. SGZ: subgranular zone

Cleaved caspase‐3 labeling was quantified in each region of interest (CA1, CA2/3, DG) for VX and IC groups (Figure 1a,b). The VX group had a significantly greater area fraction of caspase‐3 expression in CA1 (0.036 ± 0.001) and DG (0.060 ± 0.004) compared to the IC group (CA1: 0.027 ± 0.001, *p* < 0.01; DG: 0.043 ± 0.002, *p < *0.05). There was no significant difference between groups in CA2/3 (*p* > 0.05, Figure 1c). After finding cleaved caspase‐3 area fractions were elevated in CA1 and DG, the next step was to identify which cell types expressed caspase‐3 in these two regions.

### 3.3 Exercise increases caspase‐3 expression in astrocytes and radial glia‐like cells

**Figure 2:**
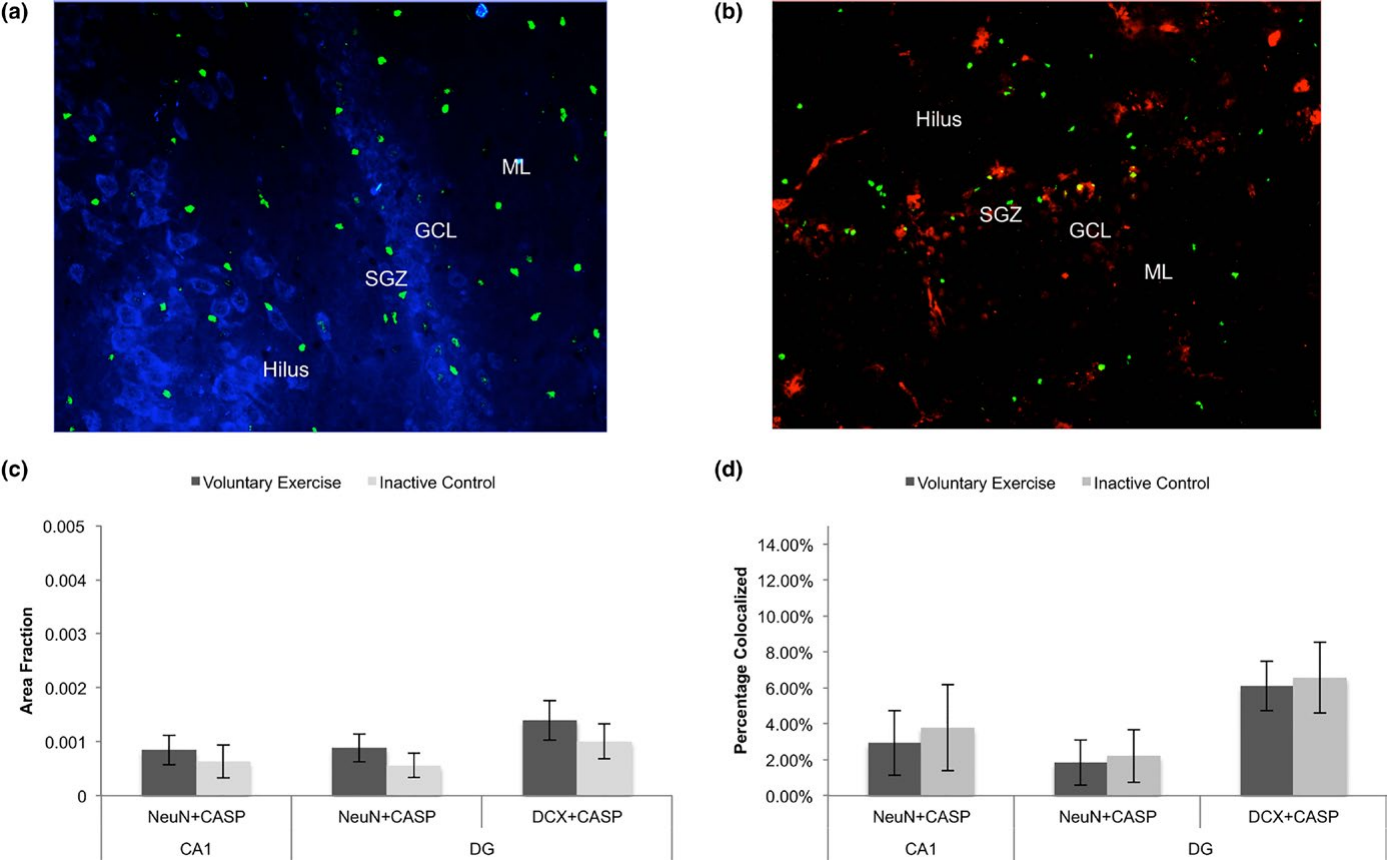
Caspase‐3 expression in neurons in CA1 and dentate gyrus (DG). (a) Immunofluorescence (IF) labeling of caspase‐3 (green) and mature neuronal marker NeuN (blue). (b) IF labeling of caspase‐3 (green) and immature neuronal marker DCX (red). (c) Between the voluntary and inactive control group, there was no difference in the area fraction of caspase‐3 (CASP) labeling colocalized with either mature neuronal marker NeuN in CA1 (*t*_(18)_ = 0.52, *p* > 0.05) or in DG (*t*_(18)_ = 0.96, *p* > 0.05). There was also no difference in CASP labeling colocalized with immature neuronal marker doublecortin (DCX) in DG (*t*_(18)_ = 0.43, *p* > 0.05). (d) In addition, out of total caspase‐3 expression, only a small percentage of caspase‐3 was colocalized in neurons labeled with either NeuN (in CA1 and DG) or DCX labeled (in DG) in voluntary exercise and inactive control groups. Values are mean ± *SEM*. SGZ: subgranular zone; GCL: Granule cell layer; ML: Molecular layer

**Figure 3:**
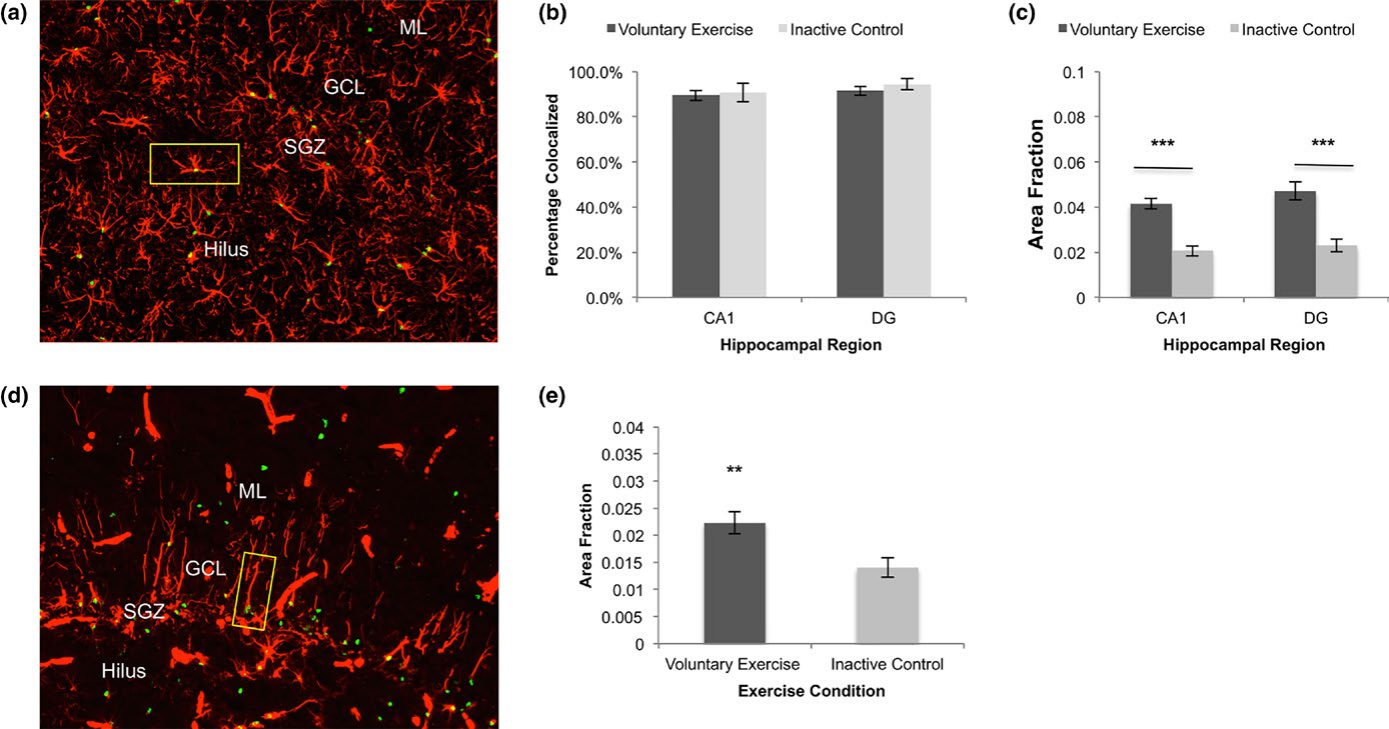
Caspase‐3 expression in astrocytes and radial glial‐like cells. (a) IF labeling of caspase‐3 (green) and astrocyte marker GFAP (red). An example of a double‐labeled cell is shown in the yellow box. (b) Out of total caspase‐3 expression, the majority of caspase‐3 was colocalized with glial fibrillary acidic protein (GFAP) positive astrocytes in voluntary exercise and inactive control groups in CA1 and dentate gyrus (DG). (c) There was a significantly greater area fraction of caspase‐3 labeling colocalized with GFAP positive astrocytes in CA1 (*t*_(18)_ = 6.36, ***p < 0.001) and DG (*t*_(18)_ = 5.14, ***p < 0.001) of the voluntary exercising group, relative to the inactive control group. (d) IF labeling of caspase‐3 (green) and radial glia‐like cell marker vimentin (red). Vimentin also labels large vessels, which were excluded from analyses. An example of a double‐labeled cell is shown in the yellow box. (e) In DG, caspase‐3 was also colocalized significantly more with vimentin‐positive, radial glia‐like cells in the voluntary exercise group, compared to the inactive control group (*t*_(18)_ = 3.02, **p < 0.01). Values are mean ± *SEM*. SGZ: subgranular zone; GCL: Granule cell layer; ML: Molecular layer

To assess which cell types expressed cleaved caspase‐3 in CA1 and DG, caspase‐3 was co‐labeled with NeuN (mature neurons), DCX (immature neurons), GFAP (astrocytes), or vimentin (RGL cells present in the SGZ, Figures 2a,b, 3a,d). Between VX and IC groups, there was no difference in the area fraction of caspase‐3 expression in the mature neurons labeled with NeuN found in CA1 (*p* > 0.05) or DG (*p* > 0.05, Figure 2c). There was also no difference between groups in the area fraction of caspase‐3 expression found in immature neurons labeled with DCX in DG (*p* > 0.05, Figure 2c). In CA1 and DG, neurons accounted for less than 10% of the total caspase‐3 expression present in both VX and IC animals (Figure 2d).

Because few neurons expressed cleaved caspase‐3, we used GFAP labeling to determine the percentage of astrocytes expressing cleaved caspase‐3 (Figure 3a). Over 90% of the caspase‐3 expression present in both IC and VX groups was in astrocytes (Figure 3b). Next, area fractions for the colocalization of caspase‐3 with GFAP positive astrocytes were quantified for VX and IC groups. In CA1 and DG, there was a significantly greater area fraction of caspase‐3 detected in the VX group (CA1: 0.038 ± 0.002; DG: 0.048 ± 0.004) compared to the IC group (CA1: 0.019 ± 0.002, *p < *0.001; DG: 0.021* ± *0.003, *p < *0.001; Figure 3c).

Because neuronal precursor, or RGL cells, in the SGZ of DG also express GFAP, it is important to understand whether these cells are in part responsible for the elevated cleaved caspase‐3 expression found in GFAP positive cells in DG following exercise. Double‐labeling for caspase‐3 and vimentin allowed for a more clear visualization of caspase‐3 in RGL cells (Figure 3d). In comparison with the IC group (0.014 ± 0.002), the VX group (0.022 ± 0.002) had significantly more caspase‐3 labeling in vimentin‐positive RGL cells (*p < *0.01, Figure 3e).

### 3.4 Caspase‐3 expression is nonapoptotic

**Figure 4:**
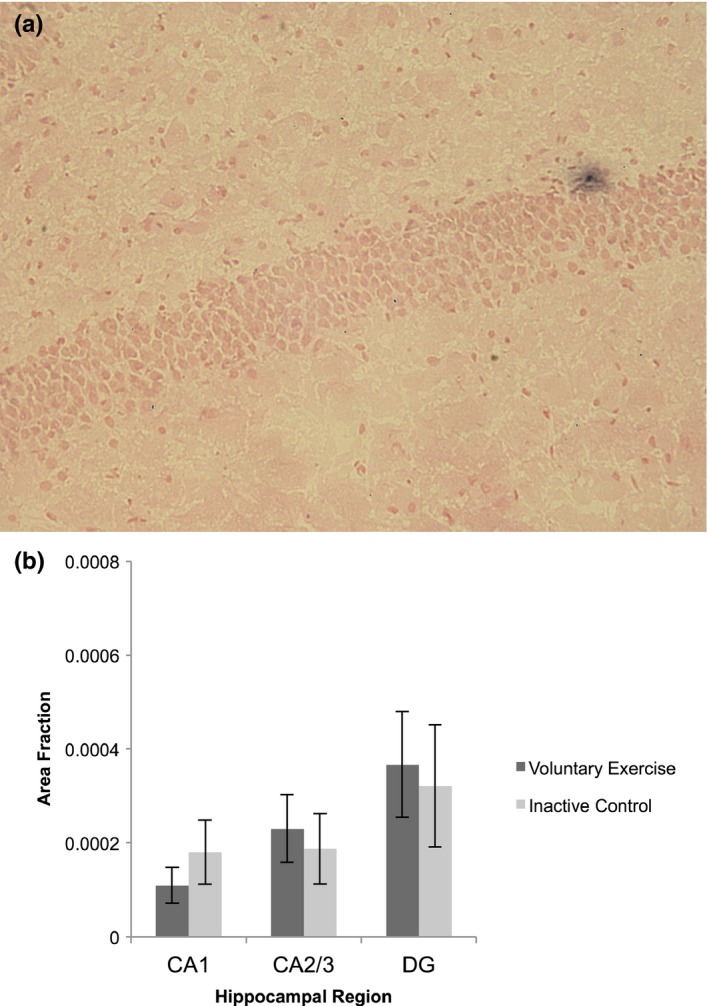
TUNEL labeling for apoptotic cells in the hippocampus. (a) Example of a TUNEL positive cell (black) in the hippocampal dentate gyrus. (b) There were no significant differences in TUNEL labeling in any region of the hippocampus (CA1: *t*_(18)_ = 0.90, *p* > 0.05; CA2/3: *t*_(18)_ = 0.41, *p* > 0.05; dentate gyrus/DG: *t*_(18)_ = 0.26, *p* > 0.05) between voluntary exercise and inactive control groups. Values are mean ± *SEM*

As a second way to quantify apoptosis, a TUNEL assay was completed and TUNEL positive cells were quantified in VX and IC groups (Figure 4a). In both VX and IC groups, remarkably few TUNEL positive cells were identified in CA1, CA2/3, or DG, and there was no difference in the area fraction of TUNEL labeling between VX and IC groups in any hippocampal region (*p* > 0.05, Figure 4b). Further, the pattern of TUNEL labeling did not mirror that of cleaved caspase‐3.

## 4 DISCUSSION

Our results demonstrate that after an acute bout of exercise, the number of cleaved caspase‐3 positive cells is elevated in hippocampal CA1 and DG regions. The cells with increased caspase‐3 expression were GFAP positive astrocytes, not neurons. In DG, RGL cells, which are also GFAP positive, contributed to this effect in exercising animals. We also found that cleaved caspase‐3 fails to fulfill its traditional role in executing apoptotic cell death, as the TUNEL assay for apoptotic cell death identified few dying cells in both VX and IC groups. In cases of apoptosis, caspase‐3 and TUNEL usually colocalize, to some degree, in the dying cell (Acarin et al., 2007; Duan et al., 2003; Fan et al., 2013; Guyenet et al., 2013; He et al., 2006; Samuel, Morrey, & Diamond, 2007; Wagner et al., 2011). For example, in the CA1 region of patients that died of cerebral infarction, immunostaining for caspase‐3 was maximal at 24 hr. TUNEL stained cells were also detectable at 24 hr, but maximal at 48–72 hr. In this experiment, there was a good correlation (*R = *0.721) found between caspase‐3 mRNA and TUNEL‐positive cells (Qi et al., 2004). In contrast, when there is minimal overlap between these two markers, the function of caspase‐3 is often nonapoptotic (Acarin et al., 2007; Fan et al., 2013; Guyenet et al., 2013; Wagner et al., 2011). In our experiment, there was no overlap between these two markers.

Specifically in astrocytes, activated caspase‐3 expression has also been detected independent of apoptosis (Acarin et al., 2007; Aras, Barron, & Pike, 2012; Guyenet et al., 2013; Noyan‐Ashraf, Brandizzi, & Juurlink, 2007; Tzeng et al., 2013). Subpopulations of astrocytes expressing nonapoptotic, activated caspase‐3 were identified in the hippocampus, cerebellum, and spinal cord of several strains of rats (Noyan‐Ashraf et al., 2007). Recently, consumption of a high fat diet increased cleaved caspase‐3 in the mediobasal hypothalamus independent of apoptotic cell death, as indicated by a lack of colocalization with TUNEL staining (Guyenet, et al., 2013). Our results add to these findings, suggesting activated caspase‐3 serves a nonapoptotic role in astrocytes, outside of major events like development or insult.

The majority of experiments involving nonapoptotic, astrocytic caspase‐3 expression have investigated it in the context of damage or development (Acarin et al., 2007; Aras et al., 2012; Tzeng et al., 2013). Following cortical excitotoxic damage, cleaved caspase‐3 expression was elevated in astrocytes near the region of infarct (Acarin et al., 2007). When comparing caspase‐3 expression to TUNEL labeling, Acarin et al.'s (2007) observations were similar to ours. Although there were some cells expressing both cleaved caspase‐3 and TUNEL, indicative of apoptosis, there was an abundance of cells expressing only cleaved caspase‐3. These nonapoptotic, caspase‐3 positive cells tended to be astrocytes, and caspase‐3 expression in these astrocytes was associated with cytoskeletal remodeling (Acarin et al., 2007). Activated caspase‐3 in astrocytes may also facilitate astrogliosis following neural damage, as inhibition of caspase‐3 diminished the expression of biochemical markers of astrogliosis (Aras et al., 2012). In another experiment, intracerebral injections of kainic acid, a model of epileptic seizure, induced excitotoxicity in the mouse hippocampus. This led to neurodegeneration and, in response, enhanced neurogenesis. In mice exposed to kainic acid, there was also a nonapoptotic increase in activated caspase‐3 expression in hippocampal astrocytes and RGL cells in the SGZ. Because there was elevated caspase‐3 expression in RGL cells *and* neurogenesis was increased, scientists next delivered a caspase‐3 inhibitor to determine whether caspase‐3 directly regulates neurogenesis. Inhibition of caspase‐3 decreased the proliferation rate of RGL cells and overall neurogenesis was impeded. Inhibition of caspase‐3 did not affect astrogliosis under these parameters (Tzeng et al., 2013).

Caspase‐3 expression in astrocytes has also been described during development. Caspase‐3 is necessary for astrocytic differentiation. In culture, inhibition of activated caspase‐3 diminished the number of differentiating Bergmann glia, a subtype of astroglia highly abundant in the cerebellum (Oomman, Strahlendorf, Dertien, & Strahlendorf, 2006), and activated caspase‐3 was elevated in the Bergmann glia of still developing cerebellar lobules, relative to the lobules that had already reached maturity (Finkbone, Oomman, Strahlendorf, & Strahlendorf, 2009). Additionally, in relation to neurogenesis, in vitro caspase‐3 activity was required for neural stem cell differentiation. When caspase‐3 activity was blocked, neurosphere differentiation was inhibited (Fernando, Brunette, & Megeney, 2005). Caspase‐3 also facilitated myoblast differentiation by inducing temporary DNA strand breaks that were selective to periods of differentiation and then rapidly repaired (Larsen et al., 2010). This evidence raises the possibility of caspase‐3 involvement in adult hippocampal neurogenesis, a process that requires differentiation and is facilitated by exercise (van Praag et al., 1999, 2005 ).

In our experiments, activated caspase‐3 expression was increased in GFAP positive astrocytes in CA1 and DG. CA1 and DG are two regions where exercise induces astrocyte structural plasticity (Ferreira et al., 2011; Komitova et al., 2005; Rodrigues et al., 2010; Saur et al., 2014; Uda et al., 2006). In CA1, astrocyte morphology changed following exercise, and processes became more complex and elongated (Saur et al., 2014). In DG, GFAP expression was also increased in the hilar region following only a few days of exercise (Ferreira et al., 2011). Growth factors that are increased with exercise, such as fibroblast growth factor or nerve growth factor, are known to facilitate astrocytic proliferation (Cragnolini, Huang, Gokina, & Friedman, 2009; Kang & Song, 2010). Saur et al. (2014) posit exercise‐induced growth factor expression may be one reason astrocytic density increases following exercise. However, it may also be of interest to investigate the role of caspase‐3 in promoting changes in astrocyte morphology after exercise, because, in other contexts, caspase‐3 has been shown to regulate changes in cell structure. Caspase‐3 regulated cytoskeletal remodeling of astrocytes following excitotoxic damage (Acarin et al., 2007), and, as mentioned previously, caspase‐3 promoted astrogliosis (Aras et al., 2012). Although astrogliosis is a defense mechanism against damage, it is also a process that induces astrocytic hypertrophy and proliferation (Colangelo, Alberghina, & Papa, 2014). Caspase‐3 is also implicated in modulating synaptic structure and function (Bravarenko et al., 2006; D'Amelio et al., 2011; Huesmann & Clayton, 2006; Kudryashova, Onufriev, Kudryashov, & Gulyaeva, 2009; Lo et al., 2015; Snigdha, Smith, Prieto, & Cotman, 2012). Activated caspase‐3 was transiently elevated in the postsynaptic terminals of the songbird auditory forebrain after repeated exposure to a novel song during habituation training. Inhibition of caspase‐3 blocked the memory of this novel song the next day (Huesmann & Clayton, 2006). Huesmann and Clayton (2006) hypothesized this effect stems from activated caspase‐3's involvement in synaptic plasticity, citing evidence that caspase‐3 targets several cytoskeletal proteins, including actin, for fragmentation (Kothakota et al., 1997; Sun, Yamamoto, Mejillano, & Yin, 1999). Together, these findings suggest a plausible role for caspase‐3 in regulating morphological changes in adult astrocytes following exercise. It will be important for future experiments to examine the effects of exercise on caspase‐3 expression in brain regions, outside of the hippocampus, that display structural plasticity in astrocytes postexercise, including the prefrontal cortex and striatum (Brockett et al., 2015; Li et al., 2005; Mandyam, Wee, Eisch, Richardson, & Koob, 2007; Tatsumi et al., 2016).

The function of activated caspase‐3 in RGL cells of the SGZ must also be considered. Exercise increased neurogenesis and the proliferation of GFAP positive cells in the hippocampal DG (Komitova et al., 2005; Uda et al., 2006; van Praag et al., 1999, 2005; Vivar, Potter, & van Praag, 2013). Inhibition of caspase‐3 activity decreased neurogenesis and the proliferation of RGL cells in a model of excitotoxic damage, implying caspase‐3 can regulate neurogenesis (Tzeng et al., 2013). However, the exact role of caspase‐3 in the regulation of neurogenesis remains unclear and difficult to identify in vivo, in part because it is expressed in both RGL cells and adult astrocytes, as in our experiment. Therefore, a global inhibition of caspase‐3 in the hippocampus would affect both types of cells. Recent experiments indicated aerobic exercise could activate quiescent RGL cells, making them enter the cell cycle more rapidly (Bouchard‐Cannon, Lowden, Trinh, & Cheng, 2018; Lugert et al., 2010). Moreover, caspase‐3 has been implicated in cell cycle regulation (Hashimoto, Kikkawa, & Kamada, 2011; Yan et al., 2001). However, adult astrocytes do not have a passive role in neurogenesis (Falk & Götz, 2017; Kempermann, 2015; Song et al., 2002; Sultan et al., 2015). Adult hippocampal astrocytes are located in close proximity with RGL cells in the SGZ and promoted neurogenesis by both increasing proliferation, and by causing more progenitors to adopt a neuronal fate (Song et al., 2002). Astrocytes also governed the survival and synaptic integration of newly birthed neurons in DG (Sultan et al., 2015). It is necessary to determine whether the function of caspase‐3 in hippocampal RGL cells and adult astrocytes is similar or fundamentally different. Currently, it seems plausible that activated caspase‐3 could regulate adult neurogenesis; however, evidence also suggests it has a more general role in modifying astrocyte structure (Acarin et al., 2007; Aras et al., 2012; Tzeng et al., 2013).

Astrocytes clearly support neurogenesis, but they are also imperative to an array of plastic events catalyzed by exercise. Exercise increased angiogenesis in the motor cortex, striatum, cerebellum, and hippocampus (Ding et al., 2006; Kerr & Swain, 2011; Li et al., 2005; Swain et al., 2003; Van der Borght et al., 2009). In the cortex and striatum, increased angiogenesis accompanied elevated astroglial proliferation (Li et al., 2005). Although not observed in the same experiment, exercise also increased angiogenesis and astrocytic proliferation in the hippocampal DG (Komitova et al., 2005; Uda et al., 2006; Van der Borght et al., 2009). In the context of exercise, the relationship between angiogenesis and astrocytic proliferation is currently only correlative; however, in other experimental paradigms, astrocytes directly promoted angiogenesis (Laterra, Guerin, & Goldstein, 1990; Scott et al., 2010; Suarez, Bodega, Rubio, Garcia‐Segura, & Fernandez, 1994; Weidermann et al., 2010; Zhai et al., 2016). In addition, astrocytes regulated vascular tone, a phenomenon that would be important for managing cerebral blood flow during exercise (Attwell et al., 2010; Lange, Gebremedhin, Narayana, & Harder, 1997; MacVicar & Newman, 2015; Mishra et al., 2016; Ogoh, Brothers, Jeschke, Secher, & Raven, 2010; Swain et al., 2003). Astrocytes also have a significant role in brain energy metabolism (Camandola & Mattson, 2017; Prebil, Jensen, Zorec, & Kreft, 2011; Riske, Thomas, Baker, & Dursun, 2017). The lactate providing fuel for the brain during exhaustive exercise is derived from glycogen in astrocytes (Matsui et al., 2017), and astrocytic lactate contributed to the maintenance of ATP levels in the brain during an endurance exercise paradigm, while ATP levels in muscles dropped. This implicates astrocytes in protecting neurons during extremely strenuous activity (Matsui et al., 2017). Exercise also increased synaptogenesis in the hippocampus, and astrocytes regulate synapse formation (Chung, Allen, & Eroglu, 2015; Dietrich et al., 2008; Farhy‐Tselnicker et al., 2017; Hughes, Elmariah, & Balice‐Gordon, 2010; Slezak & Pfrieger, 2003; Stranahan et al., 2007). In vitro*,* astrocytes secreted proteins that modulated synapse formation between hippocampal neurons (Hughes et al., 2010). Evidence reviewed clearly implicates astrocytes in the modulation of many plastic events associated with exercise. In the future, it may be interesting to determine whether astrocytic caspase‐3 supports these other forms of plasticity.

The main conclusion of our experiment is that there are an increased number of astrocytes and RGL cells in the hippocampus expressing nonapoptotic cleaved caspase‐3 after an acute bout of exercise. These findings broaden our understanding of the role of caspases in nonapoptotic processes and highlight the importance of identifying the nonapoptotic roles of caspases in astrocytic plasticity and neurogenesis. These results also emphasize the importance of determining the type of cells expressing the various factors that are elevated following exercise, as some of these factors may be expressed within astrocytes or RGL cells, rather than neuronal populations. Understanding the role of caspase‐3 in regulating adult hippocampal neurogenesis and the astrocytic plasticity that follows exercise has important clinical implications. It could aid in discerning how exercise is neuroprotective against diseases affecting the hippocampus, including dementia and Alzheimer's disease.

## CONFLICT OF INTEREST

The authors declare no conflict of interest.
